# The immune anatomy of the small intestine facilitates regulatory responses

**DOI:** 10.3389/fimmu.2026.1710639

**Published:** 2026-02-23

**Authors:** Mark W. Bodmer

**Affiliations:** Clare College, University of Cambridge, Cambridge, United Kingdom

**Keywords:** lacteal, lymph node, mesenteric, microbiota - intestinal axis, mucosal immunity, oral tolerance, small intestine

## Abstract

The intestinal immune system monitors the body’s largest interface with the environment. It must prevent adverse responses to ingested material, while protecting itself from harm, constraining the inflammatory potential of gut microbiota, and detecting pathogens. Gut immune anatomy and distribution of its microbiota may help explain how it manages these roles. The lymphatics of the jejunum and proximal ileum are anatomically independent of the rest of the gut. Mesenteric lymph nodes in these lymphatics specialize in regulatory functions. This proximal region of the small intestine, with the greatest exposure to ingested materials, is functionally sterile. The combination of lymphatic segmentation and asymmetric distribution of the microbiota allows specific functionality within regions of the small intestine which are exposed to different immune challenges. Villus lacteals draining into local lymph nodes are a route of absorption of a range of macromolecules from the small intestine enabling first-pass immunological screening of gut contents, analogous to first-pass metabolism in the liver via the portal circulation. These observations suggest an anatomical segregation enabling regulatory responses to ingested matter without compromising systemic immunity, and the potential for novel therapeutic approaches to inflammatory diseases.

## Introduction

The inner surface of the small intestine is the body’s largest area of contact with the outside world, replete with immune cells that constantly survey what passes through (1, 2). It is a sensory organ exposed to a vast quantity and diversity of foreign matter in the food we eat and associated microbial content (3). Yet we rarely suffer ill-effects of eating, other than relatively rare food allergies. The immune system recognizes and responds to these foreign substances both locally in the gut and systemically (4, 5). Lack of harm is an active process of non-response.

How does gut mucosal immunity discriminate pathogens from food and commensal microbiota? Mechanisms of non-response to ingested foreign molecules have been widely reported (6, 7), but the fundamental basis of this discrimination remains ill-defined. Existing models of immune function provide no clear explanation for molecular and cellular mechanisms of differential recognition.

Oral tolerance was first described in the early 1900s. In 1909 Besredka reported the prevention of anaphylactic responses after oral exposure to milk (8). In 1911 Wells and Osborne reported similar findings in responses to vegetable proteins (9). It has long been known that peripheral and gut mucosal immune responses to the same antigen were different, despite using the same immunological toolkit.

Most immunological discoveries over the last few decades are of systemic mechanisms of host defense, providing intricate descriptions of the network of interactions leading to effective immunity. Innate and adaptive immune pathways are bridged by pattern recognition receptors (PRRs) (10) which recognize foreign substances providing activation signals for adaptive immunity (11).

Gut mucosal immunology studies effects at epithelial barriers, with much recent focus on the microbiota, largely in the colon. Pabst and Mowat pointed out (4), “There is an important difference between tolerance to gut bacteria and tolerance to food proteins: whereas tolerance to food protein induced via the small intestine affects local and systemic immune responses, tolerance to gut bacteria in the colon does not attenuate systemic responses.” This suggests an impact on periphery immunity arising in the small intestine (12).

Gut anatomy and the distribution of the microbiota may help to explain how tolerance and protective immunity function together in the gut – recognizing food, trillions of commensal microbes, and pathogens, with differential response to each. Descriptions of gut mucosal immunity tend not to take account of the highly uneven distribution of the microbiota along the gut (13, 14). Microbial abundance in the proximal small intestine is extremely low, to the point that It is functionally sterile. It has no stable resident microbiota and deploys several non-immunological anti-bacterial mechanisms – defensins, acid, bile.

Might freedom from the need to respond to microbes in the proximal small intestine enable it to specialize in preventing inflammatory responses to ingested material? Its lymphatic anatomy suggests that it has evolved for this purpose. The lymphatic network of the jejunum and proximal ileum is anatomically independent of the GI tract above and below (15, 16). In this region which has maximum exposure to ingested materials, it appears that canonical rules of immunology are reversed, leading to non-inflammatory responses which are the basis for antigen-specific local and systemic tolerance to food. There may also be generalized anti-inflammatory responses to microbial elements in this region of the gut (17, 18).

In addition, there is a route of absorption in the small intestinal villi providing access to the local mesenteric lymph nodes. Each villus has a lacteal running through its core which is an afferent vessel leading directly to the lymph node. While lacteals have a major role in the absorption of dietary fat, their contents include a range of macromolecules that pass intact from the GI tract. This may be a route by which dietary proteins can appear in the peripheral circulation (19).

These three factors – distribution of the microbiota, anatomy of the gut lymphatics, and the lacteal route of absorption – together with regulatory function of a subset of mesenteric lymph nodes, provide a framework for viewing the complexities of gut mucosal immunology and its relationship with the periphery.

## Gut microbial abundance is unevenly distributed

The need to balance tolerance of dietary material with immunity against pathogens and containment of commensal bacteria (20) might suggest discrimination in a milieu in which they co-exist in the gut (21, 22). While mechanisms for varied responses have been reported (6, 23–25), how can they happen simultaneously in space and time?

Perhaps they do not. If the gut region in which ingested materials are the major content is separated in space and time from the region containing >99.99999% of the microbial content, then the differing immune responses to food and microbes take place in non-overlapping regions. This enables them to evolve specialized functions to mount the appropriate immune responses to their respective contents.

Studies on the impact of the gut on systemic immunity have focused on the association of changes in composition of colonic microbiota (22, 26), with effects on immunity and disease (23, 27). The microbial content of the colon is distinct from that of the small intestine, indeed, it is not clear that the proximal small intestine has a resident microbiome (13). Unlike the colon, it is free-flowing with transient microbial content from ingested material.

Microbial abundance in the upper small intestine is orders of magnitude lower than in the colon (28, 29). A recent report suggests it is as much as 8 or 9 logs lower (13). This is within the range of functional sterility. Before sensitive DNA sequencing and culture techniques for anaerobic bacteria the proximal small intestine was considered sterile (30, 31). While this turns out not strictly to be the case, abundance in the proximal small intestine is extremely low in the overall context of gut microbiota. In addition, a range of physical, chemical and biochemical protective functions in the GI tract (32) are non-immunological barriers to pathogens entering the body.

The proximal small Intestine Is the part of the body most exposed to environmental substances, to which active inflammatory responses must be prevented. With low microbial content and non-immune defenses, it may not need to wrestle the duality of protection and tolerance. Regulatory mechanisms that connect with the periphery via regional specialized lymph nodes would be consistent with Pabst and Mowat’s observations about the local and systemic effects of the small intestine (4).

## The small intestine has a discrete lymphatic network

Preventing inflammatory responses to the xenobiotic load of ingested food and microbes requires regulatory interactions in the proximal gut that lead to systemic tolerance and homeostasis. This vital role of the immune system must balance against the need for potent, and potentially self-destructive, mechanisms to protect against systemic pathogens.

**Figure 1:**
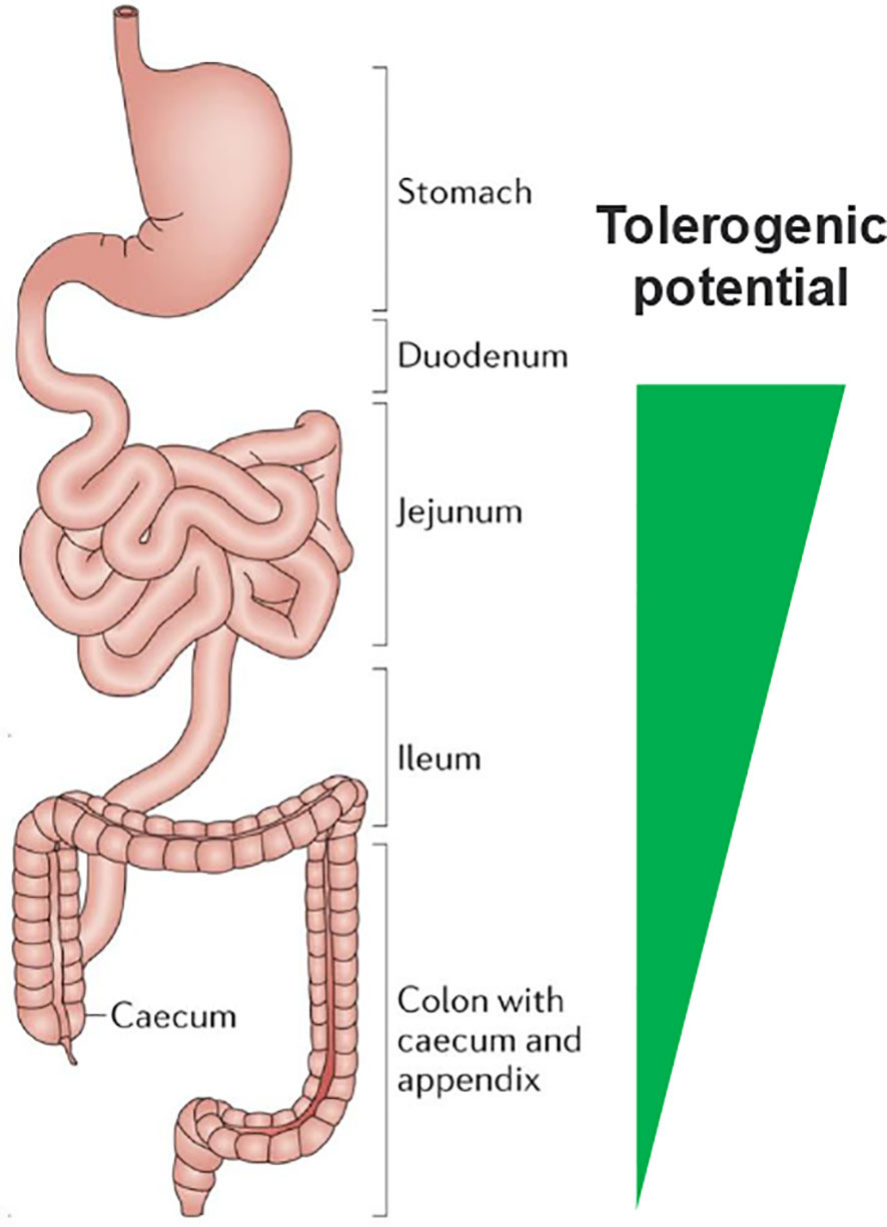
A primary role of the small intestine is to prevent systemic inflammatory reactions to food. The immune mechanisms to induce oral tolerance are concentrated in the proximal regions nearest to the stomach. Adapted with permission from Mowat, A., Agace, W. Regional specialization within the intestinal immune system. Nat Rev Immunol 14, 667–685 (2014). https://doi.org/10.1038/nri3738, Spinger Nature.

The distribution of immune cells along the gut is also non-uniform (2, 33) suggesting differential functions along its length. The proximal small intestine contains substantial numbers of CD103+CD11b+ dendritic cells (DCs) (33), a class of DC unique to the gut reported to have a role in tolerance and inflammatory homeostasis (33, 34). The abundance of these DCs in the proximal small intestine fits this model of regional immune specialization. (see **Figure 1** for a schematic view).

The distribution of lymph nodes draining the various regions of the gut has been well-described (16, 35, 36). Houston et al. (15) reported an analysis of local lymphatic drainage in the small intestine of mice using injected dye, showing site-specific uptake into individual nodes with no cross-talk to adjacent nodes. Regional segregation of immune function, with immune recognition occurring independently in small intestinal (sMLN) and colonic (cMLN) lymph nodes, enables differing immune outcomes in each. Further evidence came from tracking migratory DCs in photoconverted regions of Kaede mouse guts, revealing that the predominant CD103^+^ CD11b^+^ DC phenotype in the sMLN was relatively rare in the cMLN.

Esterhazy et al. (37) provided experimental evidence for differential spatial effects by injection of antigen into proximal and distal gut to determine the responses in the associated lymph nodes (LN). Proximal small intestinal LNs preferentially gave rise to regulatory and distal LNs to pro-inflammatory T cell responses.

Small intestinal modulation of peripheral inflammation can be harnessed pharmacologically with oral agents. Two studies reported oral administration of non-colonizing bacteria in mouse inflammation models (17, 38). The original bacterial strains were isolated from the small intestine of human donors. The bacterial preparations were non-live, did not modify the colonic microbiota and were not detected outside the gut after oral administration. Preclinical efficacy approached that of systemic dexamethasone, suggesting notable control of systemic inflammation from the small intestine. The effect was greater with formulation for release in proximal versus distal intestine (12), matching the observations of Esterhazy et al. and supporting this model of an intestinal region specializing in anti-inflammatory regulatory function contributing to systemic homeostasis.

This pharmacology was investigated in a phase 2 clinical trial in patients with mild-to-moderate plaque psoriasis (39). Meaningful efficacy was observed after oral administration of EDP1815, a non-live pharmaceutical preparation of a strain of *Prevotella histicola* (38). As in the preclinical studies, there was no host microbiome modification nor detection of EDP1815 outside the gut.

## Lymph nodes of the proximal small intestine regulate homeostasis

Maintaining homeostasis to ingested foreign molecules is a constant and intense activity of the immune system. The bulk of benign materials encountered must be many orders of magnitude higher than dangerous ones. Responses to pathogens are a critical but sporadic requirement. They are a powerful arsenal that must be constantly constrained and activated only when pathogens threaten.

Mesenteric lymph nodes (MLN) are the immunological gateway from the external environment of the gut lumen to the body’s internal environment (40). They coordinate food tolerance, non-response to commensal bacteria, and protection against pathogens. The regulatory function of MLN draining the jejunum and proximal ileum distinguishes them from MLN associated with other gut lymphoid tissues, such as Peyer’s patches (41, 42). They are the immune sensors for this relatively sterile region of the gut with high exposure to foreign material. Their anatomy and specialized mechanisms position them for the regulatory function required in response to these materials.

Ramani et al. (17) reported evidence for the role of cell trafficking through MLN in modulation of systemic inflammation by events in the gut. Lymphocyte trafficking through MLNs was blocked using antibodies against integrins α4β7 (LPAM-1) and CD62L. Blocking the MLN gateway abrogated the systemic anti-inflammatory efficacy of orally administered non-live bacteria. The anti-integrins antibodies did not block the inflammatory response.

Both proximal mesenteric lymph nodes and lymph nodes which activate protective immunity use a common set of molecular and cellular elements for differential effects. This is analogous to comparing a refrigerator with a heat pump: the component parts are essentially the same. However, the output is different depending on location and programming of the response to inputs.

Pattern recognition receptors (PRRs), first described by Janeway (10), integrate signals from diverse molecular ligands. They alert the peripheral immune system to threat (43), activating innate immunity and providing second signals required for adaptive responses. Gut mucosal immune wiring is not the same as peripheral. Receptors which are proinflammatory in the periphery can have opposite effects in the gut, e.g. Toll-like receptors TLR4 and TLR5 (44, 45). Intravenous lipopolysaccharide is pro-inflammatory. It is anti-inflammatory after oral administration (46), which can also protect against subsequent intravenous challenge (47), an innate immune equivalent of the early descriptions of oral tolerance (8, 9).

There is evidence for TLR2 involvement in oral tolerance (48). Ramani et al. (17) reported that parenteral-administered TLR2-blocking antibody abrogated systemic anti-inflammatory efficacy of orally-administered non-live bacteria, indicating a TLR2 requirement to propagate regulatory effects from gut to periphery. Tunis et al. (49) reported data that appears contradictory. Oral co-administration of a synthetic TLR2 agonist Pam3CSK4 with peanut butter tended to enhance IgE and IgA responses. Differential pathways of absorption may explain the contradiction. Peanut butter is absorbed in part through fat pathways in the lacteals which may be a route to oral tolerance (see below). Pam3CSK4 absorbed directly into the circulation would confound interpretation of the role of TLR2 by activating peripheral inflammation rather than mucosal regulation.

## Lacteals are positioned for regulatory immune surveillance

Immune responses in the gut, whether protective immunity or tolerance, require antigen sampling in the lumen and transportation across the gut wall into lymphoid compartments within the body. A mechanism for antigen capture is transcytosis across microfold (M) cells, specialized epithelial cells in the gut wall overlying lymphoid structures (50–52) which are present in the distal ileum, colon and rectum. The proximal small intestine lacks M cells (2, 53), making this antigen sampling and transport route unavailable there.

However, there is a direct route of absorption into proximal small intestinal MLN which is not available in the colon. Villi in the small intestine have a lacteal running through their core. Lacteals are lymphatic vessels named for their milky content by Italian physician, Gaspare Aselli, in 1622 (54). The primary metabolic role of lacteals is absorption of dietary fats in chylomicrons assembled in the intestinal epithelium. They flow into the local MLN and then through the thoracic duct eventually reaching the blood (55, 56).

Dietary fats are not the only materials which wind up in lacteals. As well as lipids, apolipoproteins and immune cells, chylomicrons and chyle contain an array of proteins, lipoproteins and liposaccharides (57). In 1936 Alexander et al. reported unaltered ingested egg white protein in systemic circulation of dogs (58). Egg white precipitin was found in the thoracic duct but not the portal circulation, suggesting lacteal absorption. Wang et al. (19) reported a similar phenomenon with ovalbumin in mice 73 years later. Absorption was shown to be associated with chylomicrons by co-administering long-chain triglycerides which promote formation, or Pluronic L-81, an inhibitor of lymphatic lipid transport (59).

Lacteal absorption provides a path for a wide range of molecules to pass from the gut lumen directly to the small intestinal MLN. This anatomy creates an immunological screening system for environmental substances analogous to first pass liver metabolism through the portal vein after absorption into villus capillary beds. Zawiega et al. (60) used mesenteric lymph vessel cannulation to demonstrate the wide array of molecules and cells in these fluids.

The role of lacteal absorption in immune surveillance has been reported [Nanaware et al. (61)] but its importance in homeostasis and response to the external environment has been relatively overlooked. Notably, small intestinal lymphatic vessels are common to the metabolic and immune systems. The pathway for molecules from the external environment into the body, with the sMLN as immunological gatekeepers, suggests a mechanistic link between ingested material and immune homeostasis. For example, Eckhardt and Li reported that dietary long-chain triglycerides which are absorbed in chylomicrons, enhanced oral tolerance to peanut proteins and ovalbumin. However, medium-chain triglycerides, absorbed through portal circulation rather than lacteals, aggravated anaphylactic responses to orally administered antigen (62).

## The colonic microbiome may not be vital to healthy immunity

A perspective on the gut-immune axis must consider the gut microbiome. We are symbiotic with gut microorganisms, the vast majority in the colon, widely considered as vital for proper functioning of immunity and health in a mutualistic symbiotic relationship (21, 27, 63, 64). Associations of gut microbial variation have been reported in most areas of disease (65, 66). But perhaps the relationship with the colonic microbiota is *parasitic* rather than *mutualistic* symbiosis – it grows there because it can and has inherent potential to be deleterious to the host.

This was the view of Elie Metchnikoff writing 125 years ago, “It is indubitable, therefore, that the intestinal microbes or their poisons may reach the system generally and bring harm to it. I infer from the facts that the more a digestive tract is charged with microbes, the more it is a source of harm capable of shortening life.” (67).

Metchnikoff is credited with pioneering probiotic therapy, in particular *Bacillus bulgaricus*. It turned out that *B. bulgaricus* does not survive transition through the GI tract, a general phenomenon with probiotics (68, 69). Swallowing live organisms has little impact on microbiota composition. Metchnikoff, himself, observed that effects of fermented food were as much to do with lactate and other secreted bacterial metabolites as the live bacteria.

Adult total colectomy patients remain generally healthy, despite the lack of both a colon and its microbiota. Case-control longitudinal follow-up studies show no adverse effects on general health (70). Two large cohort studies even suggest a reduced risk of cardiovascular disease (71) and type 2 diabetes after colectomy (72). This conflicts with the notion that a healthy colonic microbiome is vital to well-being. While there may be some co-evolutionary benefits of the colonic microbiome, it is not clear that it is a vital organ either in the strict sense of being necessary for life.

This creates a circular paradox. The microbes appear to have a role when present but are not required when absent. The paradox lessens if eubiosis is a parasitic rather than a mutualistic relationship. The selective benefit of water retention for animals moving out of oceans onto land required a closing sphincter at the gut terminus to allow fluid reabsorption. This created a fermenter perfectly suited for opportunistic growth of microorganisms. In healthy eubiosis the composition of the microbiota is one to which we have evolved effective physical and immunological defenses. The apparent mutualism could be the interactions which prevent the microbial content of the colon causing harm. Dysbiosis occurs when the microbial composition changes sufficiently for the defenses to become overmatched by an abundance of organisms to which we are less evolutionarily adapted. “Good” and “bad” bugs would then reflect the extent of evolutionary adaptation.

## Conclusion

This perspective shifts the focus on the gut-immune axis away from the colonic microbiota to an alternative axis arising in the small intestine. This is driven by ingested material rather than resident microorganisms, termed the small intestinal axis, Sintax (12). Four concepts help explain how the gut keeps inflammation in check as we ingest our environment:

1. The gut microbiota is largely absent in the regions of the proximal small intestine most exposed to foreign substances.
2. The jejunum and proximal ileum lymphatics are independent of the rest of the GI tract, leaving them free to induce local and systemic regulatory responses to gut contents.
3. The lymph nodes which drain the jejunum and proximal ileum mediate regulatory immunity which appears to have a central role in maintaining systemic inflammatory homeostasis in the face of exposure to ingested foreign materials.
4. The lacteals in the small intestine can absorb antigens and immunomodulatory molecules for delivery directly to regulatory lymph nodes.

The proximal small intestinal immune axis can contribute to systemic homeostasis without compromising host immunity using regionally specialized regulatory processes which are central to our well-being. These mechanisms have yet to be explored for the development of medicines, raising the possibility of a novel class of oral therapies to treat systemic inflammatory diseases.

## Data Availability

The original contributions presented in the study are included in the article/supplementary material. Further inquiries can be directed to the corresponding author.
